# Is Buffer a Good Proxy for a Crowded Cell-Like Environment? A Comparative NMR Study of Calmodulin Side-Chain Dynamics in Buffer and *E. coli* Lysate

**DOI:** 10.1371/journal.pone.0048226

**Published:** 2012-10-30

**Authors:** Michael P. Latham, Lewis E. Kay

**Affiliations:** 1 Departments of Molecular Genetics, Biochemistry and Chemistry, The University of Toronto, Toronto, Ontario, Canada; 2 Hospital for Sick Children, Program in Molecular Structure and Function, Toronto, Ontario, Canada; Concordia University Wisconsin, United States of America

## Abstract

Biophysical studies of protein structure and dynamics are typically performed in a highly controlled manner involving only the protein(s) of interest. Comparatively fewer such studies have been carried out in the context of a cellular environment that typically involves many biomolecules, ions and metabolites. Recently, solution NMR spectroscopy, focusing primarily on backbone amide groups as reporters, has emerged as a powerful technique for investigating protein structure and dynamics *in vivo* and in crowded “cell-like” environments. Here we extend these studies through a comparative analysis of Ile, Leu, Val and Met methyl side-chain motions in apo, Ca^2+^-bound and Ca^2+^, peptide-bound calmodulin dissolved in aqueous buffer or in *E. coli* lysate. Deuterium spin relaxation experiments, sensitive to pico- to nano-second time-scale processes and Carr-Purcell-Meiboom-Gill relaxation dispersion experiments, reporting on millisecond dynamics, have been recorded. Both similarities and differences in motional properties are noted for calmodulin dissolved in buffer or in lysate. These results emphasize that while significant insights can be obtained through detailed “test-tube” studies, experiments performed under conditions that are “cell-like” are critical for obtaining a comprehensive understanding of protein motion *in vivo* and therefore for elucidating the relation between motion and function.

## Introduction

A clear link has been established between protein structure, dynamics and function through studies using a wide-range of structural, biophysical and biochemical techniques [1]–[5]. For the most part, however, these studies are performed *in vitro*, using isolated proteins that have been purified to homogeneity so that the effects of the “natural biological environment” are removed. The environment in which a protein functions is a complicated heterogeneous mixture containing as many as 100 million metabolites and 2.4 million proteins per cell [6], [7]. This complex milieu can have both stabilizing and destabilizing effects on the constituent proteins. For example, the high concentrations of macromolecules can lead to increased folding propensity due to excluded volume effects, whereas, non-specific associations between macromolecules can potentially result in unfolding [8]–[15]. Additionally, the kinetics and thermodynamics of interactions can be significantly changed relative to the pristine environment of a test-tube [16]–[18]. It is clear that an in-depth picture of a protein’s structure, dynamics and function must take into account the unique properties that are found in-cell.

**Figure 1:**
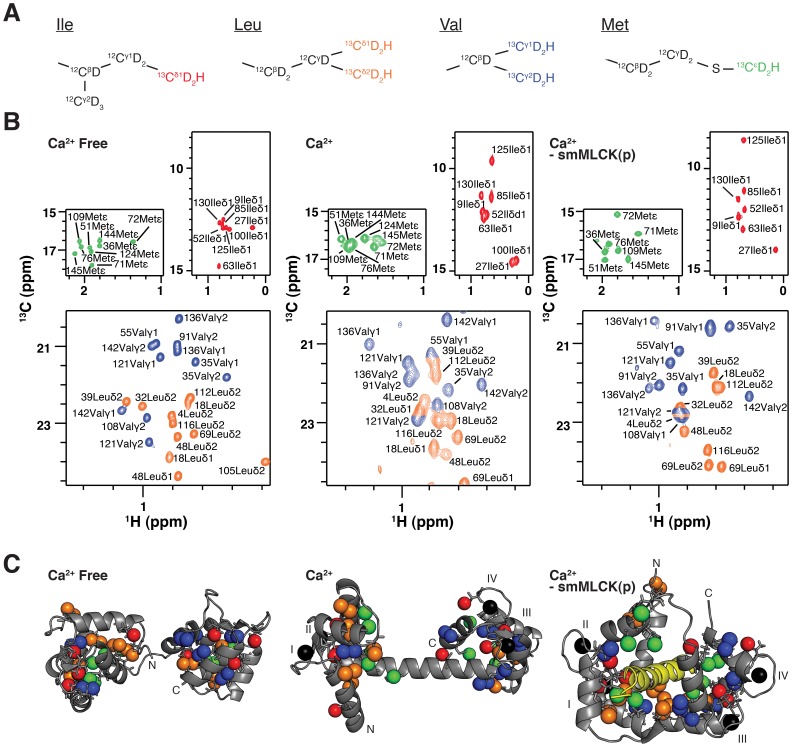
Ile, Leu, Val and Met methyl probes of CaM side-chain dynamics in *E. coli* lysate. (A) ^13^CHD_2_ labeling scheme for Ileδ1, Leuδ, Valγ and Metε methyl groups. (B) ^2^H-edited 2D ^13^C-^1^H correlation spectra of U-[^2^H],Ile-δ1[^13^CHD_2_]-, Leu,Val-[^13^CHD_2_,^ 13^CHD_2_]-, Met[^13^CHD_2_]-labeled samples of apoCaM, Ca^2+^-CaM, and Ca^2+^-CaM-smMLCK(p) in 100 g/L *E. coli* lysate. Data sets were obtained using a pulse scheme described previously for measurement of ^2^H *T_2_* relaxation times with *T* = 0 [32]. Panels show Metε, Ileδ1 and a portion of the Leuδ/Valγ region of each spectrum, with assignment of correlations as indicated. (C) Corresponding structures for the three CaM states with the ^13^CHD_2_– labeled methyl groups highlighted as spheres: Ileδ1, red; Leuδ, orange; Valγ, blue; Metε, green. An identical color scheme is used in (A) and (B). For the Ca^2+^-CaM (3CLN) [40] and Ca^2+^-CaM-smMLCK(p) (2BBM) [42] structures the four Ca^2+^ ions are shown as black spheres and the Ca^2+^ binding sites are labeled. The smMLCK(p) is in yellow for Ca^2+^-CaM-smMLCK(p). The structure of apoCaM is derived from PDB coordinates 1DMO [38].

Nuclear magnetic resonance (NMR) spectroscopy has emerged as a powerful technique for studying proteins within the biological context of the cellular environment [14], [15], [19]–[28]. While the vast majority of *in vivo* or in “cell-like” NMR studies have utilized backbone amide groups as probes of structure and dynamics, hydrophobic methyl containing side-chains could potentially also serve as valuable reporters [26], [29]. For example, NMR spectra of methyl groups have signal-to-noise ratios that are significantly higher than amide data sets [26]. Methyl containing side-chains are often localized to protein-protein interfaces, ligand binding pockets, enzyme active sites and the hydrophobic cores of globular proteins and thus provide complementary information to amide groups [30]. The importance of methyl containing residues in facilitating non-specific interactions in the cellular environment has been demonstrated by mutation of a surface hydrophobic patch on the protein ubiquitin, resulting in a significant improvement in *in vivo* NMR spectral quality [21]. This presumably reflects the elimination of transient contacts with one or more native proteins in the cell.

Here we have prepared a series of U-[^2^H], Ile-δ1[^13^CHD_2_]-, Leu,Val-[^13^CHD_2_,^ 13^CHD_2_]-, Met[^13^CHD_2_]-labeled calmodulin (CaM) samples to study methyl side-chain dynamics in a “cell-like” environment comprising 100 g/L (native protein concentration) *E. coli* lysate (referred to as lysate CaM). ^2^H-based spin relaxation experiments [31], [32] were recorded on Ca^2+^-free CaM (apoCaM in what follows) as well as Ca^2+^-loaded CaM in complex with a substrate peptide from smooth muscle myosin light chain kinase, Ca^2+^-CaM-smMLCK(p). These measurements establish that the amplitude and time-scale of fast (picosecond, ps –nanosecond, ns) methyl side-chain dynamics are very similar in aqueous buffer and under “cell-like” conditions in these two cases. In order to probe millisecond (ms) time-scale motional properties, ^1^H Carr-Purcell-Meiboom [33], [34] (CPMG) relaxation dispersion experiments [35] were recorded on apoCaM, Ca^2+^-loaded CaM (Ca^2+^-CaM) and Ca^2+^-CaM-smMLCK(p) protein states. Striking differences in ms motions were observed for buffer and lysate apoCaM, resulting from sampling of a metal bound intermediate in the lysate environment. Similarly, large differences in dispersion profiles were noted for buffer and lysate Ca^2+^-CaM, reflecting transient CaM interactions with native proteins in the lysate. By contrast, relaxation dispersion experiments indicate very much reduced ms time-scale dynamics for lysate Ca^2+^-CaM-smMLCK(p) relative to either lysate apoCaM or lysate Ca^2+^-CaM. Our results highlight both the similarities and the differences in motional properties of CaM in the two environments and therefore the importance of extending studies of protein structure and dynamics beyond the test-tube.

**Figure 2:**
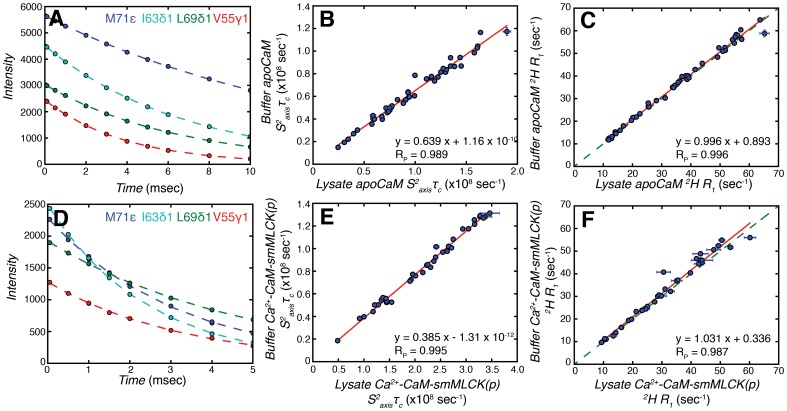
^2^H spin relaxation rates for methyl groups in lysate apoCaM and lysate Ca^2+^-CaM-smMLCK(p). (A and D) Representative ^2^H *R_2_* decay curves for (A) lysate apoCaM and (D) lysate Ca^2+^-CaM-smMLCK(p). (B and E) Linear correlation plots of/values for methyl groups in (B) apoCaM and (E) Ca^2+^-CaM-smMLCK(p) dissolved in either aqueous buffer (Y-axis) or lysate (X-axis). The best-fit line to the data is shown in red along with Pearson’s correlation coefficient, *R_p_*. (C and F) Linear correlation plots of ^2^H *R_1_* relaxation rates measured for (C) apoCaM and (F) Ca^2+^-CaM-smMLCK(p) in aqueous buffer (Y-axis) and lysate (X-axis). The green dashed line is y = x, while the red line is obtained from linear regression.

## Results and Discussion

### Methyl Labeling Strategy and NMR Spectra

Figure 1A illustrates the methyl side-chain labeling strategy utilized in this study in which U-[^2^H], Ile-δ1[^13^CHD_2_]-, Leu,Val-[^13^CHD_2_,^13^CHD_2_]-, Met[^13^CHD_2_]-labeled proteins were produced. This labeling scheme was chosen for several reasons. First, ^13^C-^1^H correlation spectra of recombinant, labeled CaM recorded in the complex, unlabeled lysate mixture could be simplified by editing through the attached deuterons, ensuring that only cross peaks from CaM are observed. Second, transverse relaxation of the deuteron is rapid (on the order of several ms in the applications considered here) and the ^2^H gyromagnetic ratio is small so that there is negligible contribution to magnetization decay from chemical exchange [36]. We show below that this is critically important for the extraction of accurate ps-ns dynamics parameters for lysate CaM, where chemical exchange contributions to the transverse relaxation of spin ½ nuclei can be significant. Third, the ^13^CHD_2_ labeling scheme is well suited for recording ^1^H CPMG relaxation dispersion profiles as a probe of ms time-scale motions [35].

**Figure 3:**
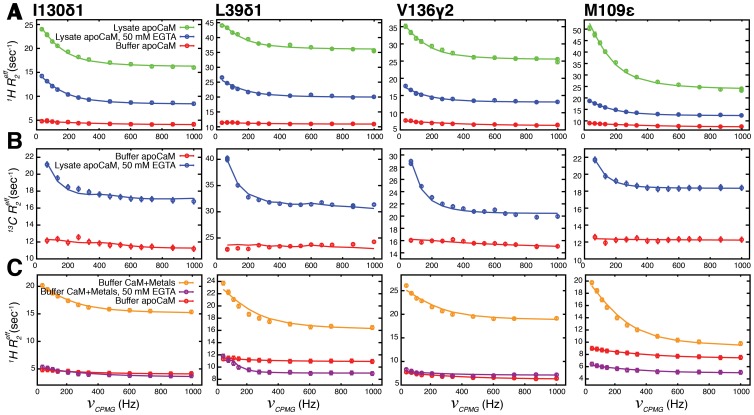
Chemical exchange in lysate apoCaM. Representative CPMG relaxation dispersion profiles for methyl groups I130δ1, L39δ1, V136γ2 and M109ε. (A) ^1^H CPMG dispersion curves for lysate apoCaM (green, 100 g/L *E. coli* proteins), lysate apoCaM, 50 mM EGTA (blue) and buffer apoCaM (red). (B) ^13^C CPMG dispersion profiles for buffer apoCaM (red) and lysate apoCaM, 50 mM EGTA (blue). (C) ^1^H CPMG dispersion curves for buffer, CaM with (orange) or without (red) metals found in *E. coli* lysate, or with metals and 50 mM EGTA (purple). Solid lines are the result of global fits of the data to a two-site exchange model.

Recombinant, U-[^2^H], Ile-δ1[^13^CHD_2_]-, Leu,Val-[^13^CHD_2_,^ 13^CHD_2_]-, Met[^13^CHD_2_]-labeled samples of CaM (1 mM) were over-expressed and purified from *E. coli* and then added to concentrated, unlabeled (protonated) *E. coli* lysate (final lysate protein concentration of 100 g/L). In the following study we prefer to mimic “in-cell” conditions using *E. coli* lysate rather than perform “true” *in vivo* experiments in which the protein of interest is either over-expressed in *E. coli* [15], [24], [26], [28], [29] or injected into Xenopus oocytes [19], [21], [23]. The use of lysate samples obviates the constant need to verify that the recorded signal derives from intracellular rather than extracellular protein, the latter due to leakage from dead or damaged cells [37]. In this regard lysate samples have proven to be stable over periods of several months and samples can be used repeatedly provided that the lysate is periodically mixed to remain homogeneous. In addition, high quality, quantitative NMR data can be recorded on lysate CaM samples since reasonable concentrations of CaM can be added; the lower CaM concentrations typical of over-expression or injection techniques and the short amounts of measurement time prior to protein leakage are significant obstacles to “in cell” NMR. Finally, lysate samples are easily manipulated by including additives in ways which are much more difficult in the case of *in vivo* experiments (see below).

**Figure 4:**
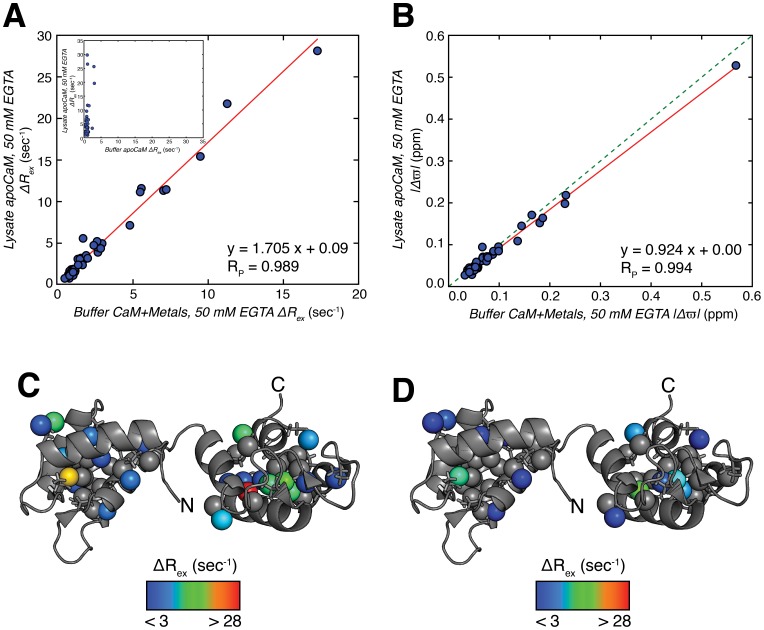
A major contribution to chemical exchange in lysate apoCaM from metal binding. (A) Linear correlation plot of ^1^H Δ*R_ex_* values calculated from dispersion profiles recorded on lysate apoCaM, 50 mM EGTA (Y-axis) and buffer CaM+metal, 50 mM EGTA (X-axis; metals added to approximate concentrations in *E. coli* lysate). The best-fit line is shown in red. Inset shows a poor correlation between lysate apoCaM, 50 mM EGTA (Y-axis) and buffer apoCaM (X-axis), providing further evidence that dispersions in lysate derive from metal. (B) Linear correlation plot as in (A) of |Δ*ϖ*| values extracted from fits of ^1^H CPMG dispersion profiles to a model of two-site chemical exchange. (C and D) Structures of apoCaM (1DMO) [38] color coded according to methyl ^1^H Δ*R_ex_* values as measured from dispersion profiles recorded on lysate apoCaM, 50 mM EGTA (C) and buffer CaM+metal, 50 mM EGTA (D). Methyl groups with ^1^H Δ*R_ex_* <3 sec*^−^* ^1^ are colored gray.

Figure 1B shows Metε, Ileδ1 and Leuδ/Valγ regions from 2D ^2^H-edited ^13^C-^1^H correlation spectra recorded on apoCaM, Ca^2+^-CaM and Ca^2+^-CaM-smMLCK(p), each in 100 g/L *E. coli* lysate. High quality correlation maps are obtained for both apoCaM and Ca^2+^-CaM-smMLCK(p) while a noticeably poorer quality spectrum is derived from Ca^2+^-CaM. These differences can be understood in terms of the structures of each state, as illustrated in Figure 1C. For example, in apoCaM the majority of the methyl side-chains are buried inside the hydrophobic cores of the N- and C-terminal domains [38], [39], leading to high quality correlation spectra. Upon binding of four Ca^2+^ ions, large structural changes occur to CaM resulting in the exposure of many of the methyl side-chains, especially those of the nine Met residues in the protein (green spheres in Figure 1C) [40]. This rearrangement is responsible for the large chemical shift differences between corresponding peaks in the apoCaM and Ca^2+^-CaM spectra [41]. It also leads to significant peak broadening due to transient non-specific interactions with native proteins in the lysate, as is discussed below. Upon smMLCK peptide binding, the majority of the methyl side-chains are again sequestered within the hydrophobic core of Ca^2+^-CaM-smMLCK(p) [42] and a high quality correlation spectrum is recovered.

**Figure 5:**
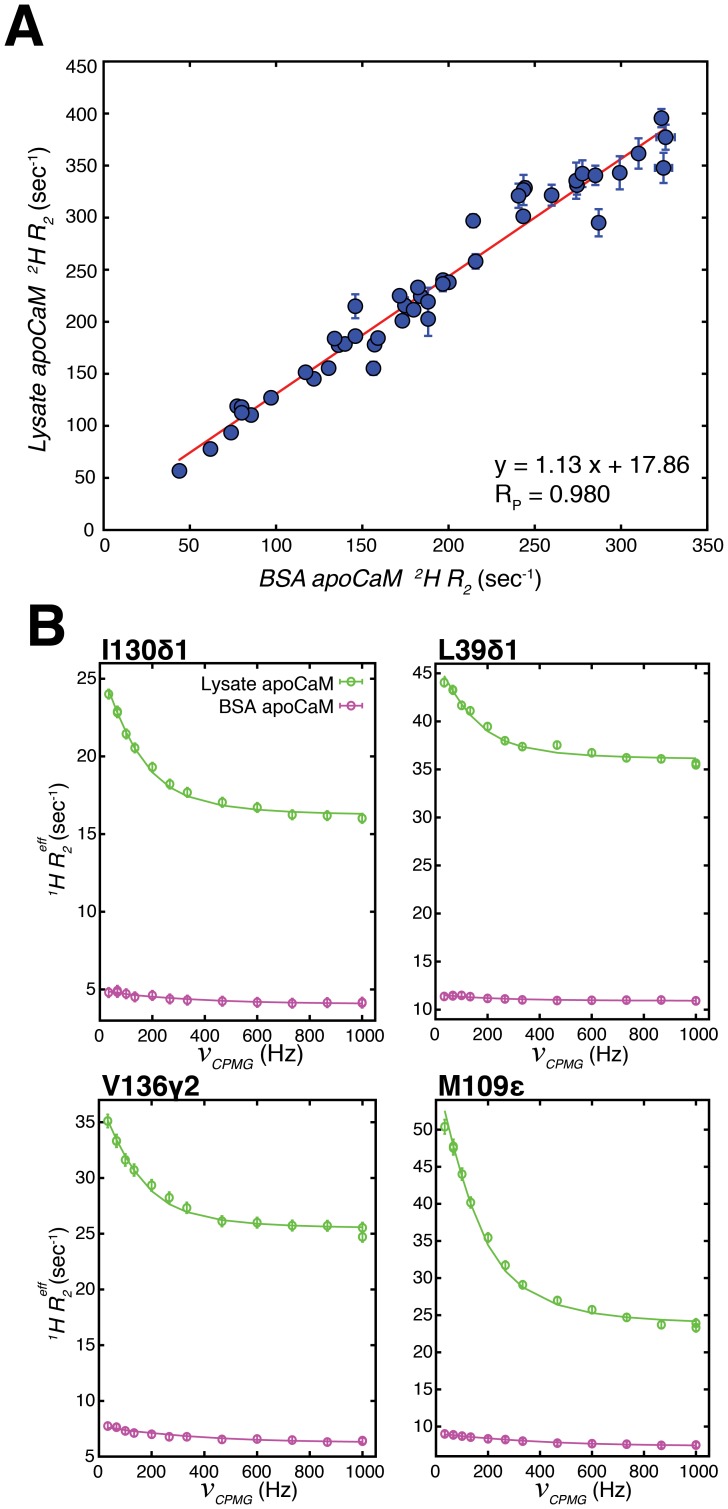
Chemical exchange in lysate apoCaM is not due to crowding. (A) Linear correlation plot of methyl ^2^H *R_2_* relaxation rates from lysate apoCaM (Y-axis) and BSA apoCaM (200 g/L BSA, X-axis). Best-fit line in red. (B) Representative ^1^H CPMG relaxation dispersion profiles for BSA apoCaM (pink) and lysate apoCaM (green). The solid lines derive from global fits of dispersion profiles to a two-site exchange model.

### Fast Time-scale Methyl Side-chain Dynamics

^2^H longitudinal, *R_1_*, and transverse, *R_2_*, relaxation rates [31], [32] were measured for the Ile, Leu, Val and Met methyl groups of apoCaM and Ca^2+^-CaM-smMLCK(p), both in aqueous buffer and in 100 g/L *E. coli* lysate. Corresponding data were not obtained presently for Ca^2+^-CaM dissolved in lysate as the quality of the relaxation spectra was significantly worse (see below). Figures 2A and 2D show representative decay curves used to determine ^2^H *R_2_* relaxation rates for a number of methyl groups in lysate apoCaM (Figure 2A) and lysate Ca^2+^-CaM-smMLCK(p) (Figure 2D). Similarly high quality decay curves were obtained from *R_1_* measurements. Our choice of using the ^2^H as a probe of ps-ns time-scale dynamics is based on the fact that its relaxation is dominated by the quadrupolar interaction, thus minimizing contributions from chemical exchange to transverse relaxation rates [36]. This is a particular concern for studies of CaM in lysate where we show in what follows that chemical exchange can be particularly pervasive leading to significantly elevated transverse relaxation rates for spin ½ nuclei (such as ^1^H, ^15^N or ^13^C) and thus erroneous extracted dynamics parameters, unless special care is taken [43]. By contrast, ^2^H relaxation rates are much less sensitive to such effects, as illustrated below.

**Figure 6:**
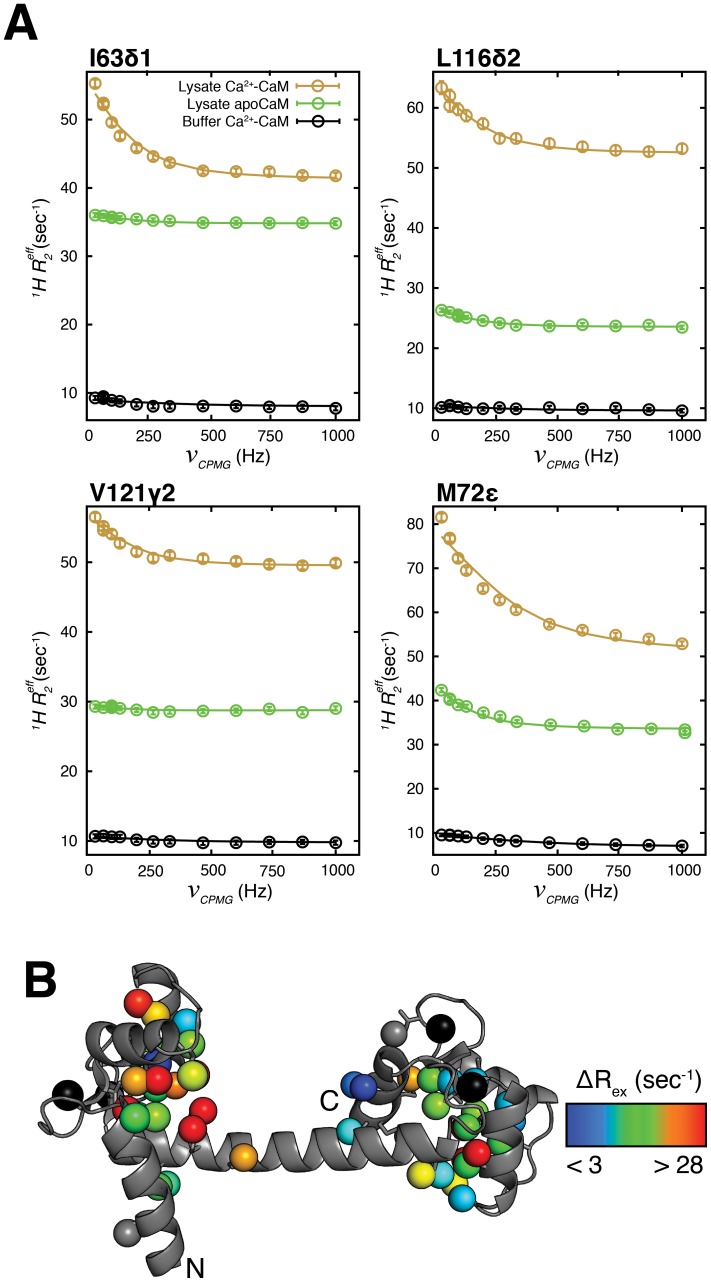
Pervasive chemical exchange in lysate Ca^2+^-CaM. (A) Representative ^1^H CPMG relaxation dispersion profiles from I63δ1, L116δ2, V121γ1 and M72ε methyl groups of buffer Ca^2+^-CaM (black) and lysate Ca^2+^-CaM, 6 mM Ca^2+^ (brown). For comparison, dispersion curves are also shown for lysate apoCaM (green). (B) Structure of Ca^2+^-CaM (3CLN) [40] with methyl groups color coded according to ^1^H Δ*R_ex_* values. Methyl groups with ^1^H Δ*R_ex_* <3 sec*^−^* ^1^ are colored gray. The four Ca^2+^ ions are shown as black spheres.

As has been described in detail previously, for a ^13^CHD_2_ group and under the conditions of our experiments, the effective ^2^H *R_2_* and *R_1_* relaxation rates are related to motional parameters via the relations, [31], [32]

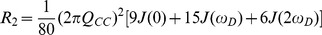
(1)and



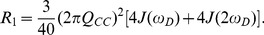
(2)In its simplest form, the spectral density function, 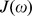, is defined as [44], [45]

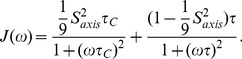
(3)


In eqs 1,2 *Q_cc_* = 167 kHz is the ^2^H quadrupolar coupling constant [46], τ*^−1^ = τ_C_^−1^+ τ_f_^−1^*, *τ_C_* is the rotational correlation time assuming isotropic tumbling, *τ_f_* is a time constant for ps time-scale C-D bond vector fluctuations that include methyl rotation and 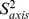 is the square of an order parameter that describes the amplitude of motion of the methyl rotation axis. In a “typical” analysis, values of *τ_f_* and 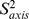 are extracted from fits of *R_1_*, *R_2_* rates to the model described above, eqs 1–3, assuming a value for *τ_C_* that is obtained from backbone ^15^N spin relaxation experiments [36], [47]–[49]. Here we have taken a different strategy that avoids complications that emerge from a potentially erroneous *τ_C_* value resulting from ^15^N relaxation rates “contaminated” by chemical exchange.

**Figure 7:**
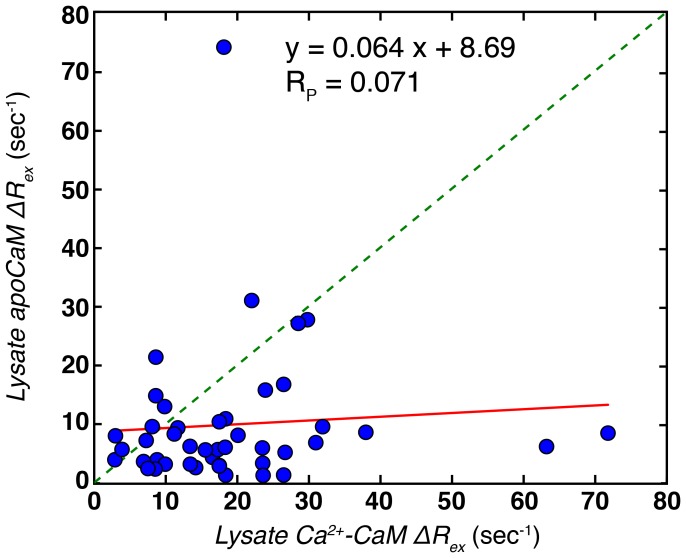
Slow time-scale dynamics in lysate Ca^2+^-CaM do not derive from metal binding. Linear correlation plot of ^1^H Δ*R_ex_* values from lysate apoCaM (Y-axis) and lysate Ca^2+^-CaM with 6 mM Ca^2+^ (X-axis). The red line derives from linear regression of the data.

Since our goal presently is to compare ps-ns time-scale dynamics of different states of CaM in buffer or lysate we will not attempt to separate 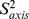 and *τ_C_* which would require an accurate value for *τ_C_* (assuming of course that the overall tumbling can be described by a single correlation time in the first place). Rather, from measurements of ^2^H *R_1_* and *R_2_* rates we show below that accurate per-residue 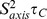 values can be isolated and can then be compared between samples to establish similarities in amplitudes of motion. Further, as described previously [31], in the macromolecular limit that is appropriate in the studies here (see below) a per-residue comparison of ^2^H *R_1_* rates can be used to evaluate the similarity in *τ_f_* values between corresponding methyl sites in different samples.

In the limit that 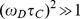, 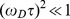 eqs 1,2 can be rewritten as
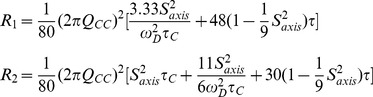
(4)and the errors introduced by these simplifying assumptions are small. For example, for *τ_C_* = 4 ns, 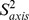 = 0.5, *τ_f_* = 20 psec, 14.0T errors of 8.6 and 3.3% are obtained for the R_1_, R_2_ rates, respectively, that decrease to 0.33 and 0.02% for *τ_C_* = 15 ns. As 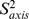 increases the errors become slightly larger (*τ_C_* = 4 ns, 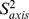 = 1.0, *τ_f_* = 20 psec, 14.0 T, errors of 11 and 3.5%; *τ_C_* = 15 ns, 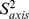 = 1.0, *τ_f_* = 20 psec, errors of 0.5 and 0.03%), but are still negligible for applications involving the size of proteins considered here (see below). Similarly small errors are obtained over the range of *τ_f_* values typically observed (10 ps ≤ *τ_f_* ≤100 ps). Combining expressions for *R_1_* and *R_2_* above one obtains



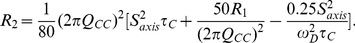
(5)It is straightforward to show that the final term in eq 5 is small and can be neglected for any reasonable values of dynamics parameters. For *τ_C_* = 2 ns, 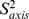 = 0.5, *τ_f_* = 20 psec, 14.0 T, omission of this term introduces an error of ∼ 8.0% that decreases to 0.1% for *τ_C_* = 20 ns. In general, errors increase very slightly as a function of 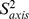 and for decreasing values of *τ_f_* and decrease with increasing *τ_C_* but do not exceed 10% even for *τ_C_* = 2 ns.

Tjandra *et al* have analyzed ^15^N backbone relaxation experiments recorded on apoCaM in aqueous buffer [50] and Lee *et al* have carried out similar analyses for Ca^2+^-CaM-smMLCK(p) [51]. Taking into account the differences in temperatures between their work and that reported here, as well as the different solvent systems used (100%D_2_O *vs.* 90%H_2_O, 10%D_2_O), the N- and C-terminal domains of apoCaM are predicted to tumble with correlation times of ∼17 and ∼14 nsec, respectively, under our conditions (18°C), while both domains of Ca^2+^-CaM-smMLCK have predicted *τ_C_* values of ∼15 nsec. Correlation times will, of course, be higher in lysate (see below). The final term in eq 5 contributes no more than 0.3% to *R_2_* and can therefore be neglected in our work here. Thus, from measurement of both *R_2_* and *R_1_* values it is possible to obtain accurate values of the product 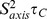.

Figure 2B shows the linear correlation plot of 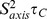 values for methyl groups of apoCaM in buffer (Y-axis, buffer apoCaM) and in 100 g/L *E coli* lysate (X-axis, lysate apoCaM) with the corresponding plot for Ca^2+^-CaM-smMLCK(p) indicated in panel E. An excellent correlation is obtained for both datasets, with Pearson’s correlation coefficients of 0.989 and 0.995, respectively, indicating that for both states of CaM 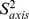 values are the same in lysate and aqueous buffer. Moreover, the excellent correlations observed in Figures 2B,E also establish that the methodology outlined above for extracting values of 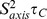 is robust, as expected based on anticipated errors described above. Not surprisingly there is an increase in *τ_C_* for samples dissolved in lysate relative to buffer that manifests in a slope different from unity. Despite the fact that very similar concentrations of lysate are used for apoCaM and Ca^2+^-CaM-smMLCK(p) samples, the relative increase in tumbling time is significantly more for peptide bound CaM (lysate *vs* buffer), reflecting the differences in shapes of the two molecules.

**Figure 8:**
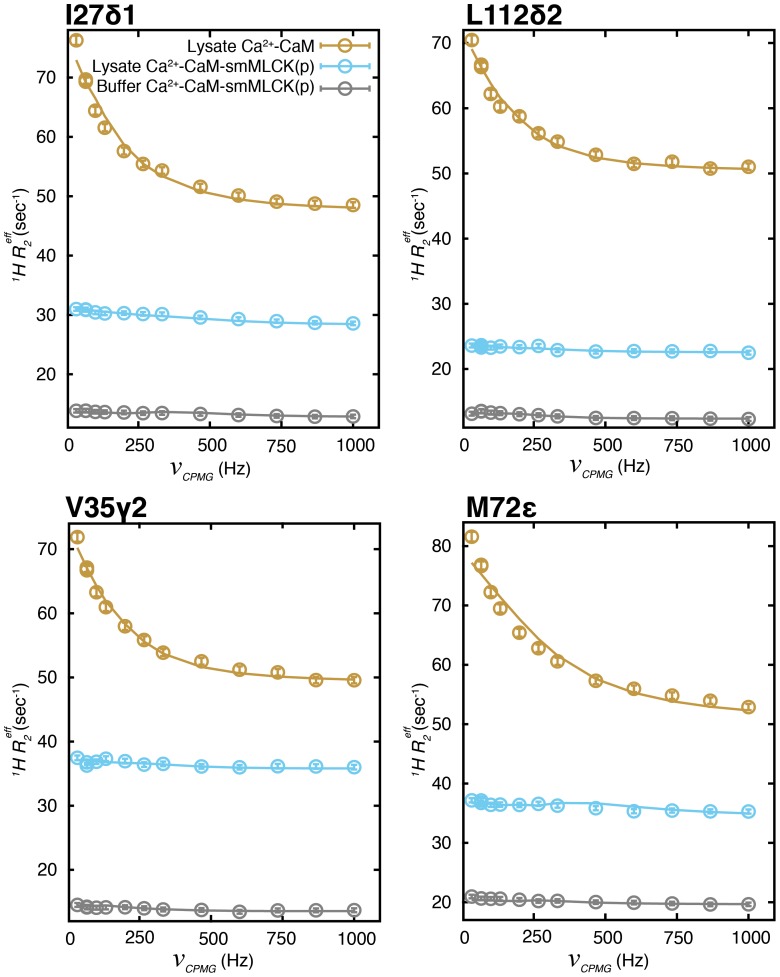
Chemical exchange in lysate Ca^2+^-CaM is eliminated through the addition of smMLCK(p). Representative ^1^H CPMG relaxation dispersion curves for methyl groups I27δ1, L112δ2, V35γ2 and M72ε of lysate Ca^2+^-CaM (brown), lysate Ca^2+^-CaM-smMLCK(p) (light blue) and buffer Ca^2+^-CaM-smMLCK(p) (grey). Solid lines derive from fits of profiles to a two-site global exchange model.

Figures 2C and 2F plot ^2^H *R_1_* values for methyl groups of CaM dissolved in buffer and lysate and a very high degree of correlation is obtained. Simulations establish that for *τ_C_* values on the order of 15 and 30 ns, as is the case for CaM dissolved in buffer and lysate, respectively, an agreement between *R_1_* rates of the sort illustrated here with a slope of 1 is only possible if the *τ_f_* values for a given methyl group are very similar for both buffer and lysate solvents.

In summary, the results presented above establish that, at least in the context of a simple model of protein ps-ns time-scale dynamics and for apoCaM and Ca^2+^-CaM-smMLCK(p) samples, motional parameters (in this case 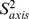, *τ_f_*) are unaffected by lysate. Previous studies have indicated that protein dynamics may be slaved to solvent motion and that perturbations to solvent can be ‘felt’ within the protein hydrophobic core [52]–[56]. In this context our results suggest that the properties of the solvent surrounding CaM in lysate and aqueous buffer are similar – at least with regard to coupling to fast time-scale protein dynamics - consistent with ^2^H *R_2_* measurements of D_2_O in living bacterial cells performed by Persson and Halle, where it was determined that ∼85% of cell water has bulk-like dynamics [57].

### Chemical Exchange in Lysate apoCaM

Results described in the previous section indicate that ps-ns time-scale side-chain dynamics of methyl containing residues in apoCaM and Ca^2+^-CaM-smMLCK(p) are very similar. In an effort to extend our study to additional time-scales we have recorded ^1^H- and to a lesser extent ^13^C-CPMG [35], [58] relaxation dispersion experiments that probe ms motions. As described above, the methyl side chains of apoCaM are predominantly buried within the hydrophobic cores of the N- and C-terminal domains [38], [39] and are thus unavailable for interaction with the natural proteins of the *E. coli* lysate. Our initial assumption was, therefore, that there would be little difference between buffer and lysate apoCaM with respect to slow timescale dynamics, as probed by methyl groups. Representative ^1^H CPMG dispersion profiles, Figure 3A, illustrated for 4 residues of apoCaM show that this is, indeed, not the case. For many of the methyl groups in the protein significant dispersion profiles were measured for lysate apoCaM (green circles), indicating ms timescale conformational exchange. Excellent fits (solid lines) of methyl dispersion data recorded at 14.0 T were obtained using a two-site chemical exchange model [59], with a global exchange rate of 880±30 s*^−^* ^1^ between interconverting conformations (see Materials and Methods). By contrast, the corresponding curves for buffer apoCaM (red) showed little dependence on the time between successive pulses of the CPMG element, δ, (ν*_CPMG_* = 1/(2δ)) and therefore much less evidence for the same ms exchange process (compare red and green curves).

In order to establish the origin of the exchange in the lysate environment we added 50 mM EGTA to the lysate apoCaM sample and repeated the ^1^H CPMG experiments (blue). Dispersion profiles were significantly attenuated but nevertheless were not quenched, Figure 3A (compare green and blue). These results are not artifactual. ^13^C CPMG dispersion profiles, recorded on U-[^2^H], Ile-δ1[^13^CH_3_]-, Leu, Val-[^13^CH_3_,^ 12^CD_3_]-, Met[^13^CH_3_]-labeled samples of apoCaM in buffer and lysate containing EGTA (Figure 3B), were similar to those recorded with the ^1^H experiments. The partial attenuation of exchange effects upon addition of chelator provides strong evidence that metal binding to apoCaM contributes at least somewhat to the CPMG dispersion profiles. To investigate this more fully we have prepared a sample of buffer apoCaM to which has been added metals in concentrations corresponding to those found in *E. coli* lysate (free + bound metal), as determined by atomic absorption analysis (see Materials and Methods). No attempt has been made to compensate for the fact that some of the metals will be sequestered by proteins in the lysate, as the fraction bound is unknown. Large dispersion profiles are observed, Figure 3C (orange) that are of a similar magnitude to those recorded for the lysate, apoCaM sample (Figure 3A, green). These dispersions can be attenuated significantly, but not eliminated, by addition of 50 mM EGTA, Figure 3C purple, as observed upon addition of chelator to lysate, apoCaM (Figure 3A, blue).

The effect of metal can be quantified more fully by comparing the magnitudes of ^1^H dispersion profiles, 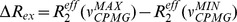, recorded on samples of lysate apoCaM, EGTA and buffer CaM+metal, EGTA. Figure 4A shows an excellent correlation between 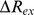 values, consistent with similar exchange processes in both cases. By contrast, a poor correlation is obtained when values from samples of lysate apoCaM, EGTA and buffer apoCaM (*i.e*., no added metal) are compared (Figure 4A, inset) that provides further evidence that the dispersions observed in the lysate derive largely from metal exchange (see below). Dispersion profiles for lysate apoCaM, EGTA and buffer CaM+metal, EGTA have been fitted to a model of two-site exchange and extracted absolute values of chemical shift differences, 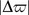 (ppm), are in good agreement, Figure 4B, as would be expected for exchange processes sharing a common mechanism. Fitted values of *k_ex_* are similar between samples, but not identical (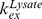 =  600±55 s*^−^* ^1^, 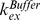 =  750±90 s*^−^* ^1^), reflecting potential differences in metal concentrations as well as differences in on/off rates between crowded lysate and buffer environments. The fitted values of 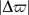 and *k_ex_* also rationalize the linear relationship of Figure 4A, where for the majority of residues 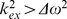. In this limit it can be shown [60] that a plot of *vs* 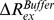 would be expected to be approximately linear assuming that a two-site exchange mechanism is valid (*i.e*., binding occurs to predominantly a single, high affinity site) with 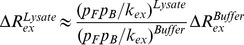, where *p_F_* and *p_B = _*1−*p_F_* are the fractional populations of metal free and metal bound states. Note that while an excellent correlation of Δ*R_ex_* values is predicted in this case, the slope of the correlation plot (
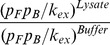
) can differ from unity. From the fitted exchange parameters 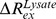 is calculated to be greater than 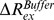, as observed (Figures 3A,C and 4A), that is not inconsistent with metal exchange playing a major role in the exchange process in both cases.

Figures 4C, D show that the large Δ*R_ex_* values from lysate and buffer+metal CaM samples (with EGTA) localize to the same sets of residues. Most of the methyl groups with substantial Δ*R_ex_* can be found in the C-terminal domain and include V108Cγ1, V91Cγ1, I100Cδ1 and L105δ1/δ2, located in Ca^2+^-binding site-III, which has the highest affinity for Ca^2+^ [61]. It has previously been demonstrated that site-III is the first to bind Ca^2+^, [61] so that at sub-stoichiometric Ca^2+^ concentrations (recall that 50 mM EGTA has been added) exchange between free and bound conformers would be expected in this region.

These results clearly establish that metal exchange in lysate apoCaM plays a very significant role in the ms dynamics that are observed and that contributions from interactions with endogenous *E. coli* proteins in lysate are at most only very minor. Indeed, the correlations observed in Figures 4A,B would not be obtained otherwise. As an aside it appears that 50 mM EGTA is not sufficient to sequester all of the metal and quench the exchange process. We were interested to see if there is an additional component to the dispersions that might derive from macromolecular crowding. To this end we prepared a sample of apoCaM in buffer containing 200 g/L BSA (referred to as a BSA apoCaM sample). This concentration of BSA results in a similar macroscopic viscosity to that obtained with 100 g/L lysate, as established by a comparison of ^2^H *R_2_* values for CaM measured in lysate and BSA samples, Figure 5A. In the macromolecular limit 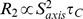, eq 5, and the similar *R_2_* values obtained in the two samples imply, therefore, comparable CaM tumbling times and hence similar solution viscosities. Figure 5B compares ^1^H CPMG dispersion profiles of lysate apoCaM and BSA apoCaM samples. Profiles from BSA apoCaM are very much smaller than for lysate apoCaM so that if crowding does in fact contribute to ms dynamics it does so only in a very minor way.

Interestingly, fluorescence experiments on and computer simulations of apoCaM in the crowded environment of the inert glucose polymer Ficoll indicate that crowding (i) lowers the inter-domain distance (see Figure 1C) producing a second population of more compact structures, (ii) increases the flexibility of the C-terminal domain by weakening tertiary interactions and (iii) partially exposes hydrophobic patches responsible for substrate binding [62], [63]. ^13^C-^1^H correlation spectra of apoCaM in both lysate and BSA are very similar to those recorded in aqueous buffer, suggesting little conformational change or alternatively one that involves only a very small population of molecules that is below the detection threshold of our experiments. Moreover, we see no differences in ps-ns internal dynamics or in ms time-scale dynamics between CaM samples in buffer or 200 g/L BSA, suggesting that if there is a change in flexibility it involves frequencies that are distinct from those monitored in the present set of experiments or that the changes do not involve methyl containing residues.

Finally, Figure 5A illustrates an important point, emphasized earlier in the text, concerning the use of deuterium spin relaxation. Despite the fact that there are significant exchange contributions to ^13^C and ^1^H linewidths in spectra of apoCaM in lystate, as established by relaxation dispersion experiments (see Figure 3), these impact negligibly on ^2^H *R_2_* rates so that accurate values are obtained. Note that the excellent correlation in Figure 5A could not be realized if the ms dynamics in the lysate apoCaM sample were to significantly influence ^2^H *R_2_* rates. In this context we have found that ^13^C transverse relaxation rates of methyl groups in lysate apoCaM (measured using ^13^CHD_2_ probes) are consistently larger than expected based on ^2^H measurements. As discussed previously, the deuteron is an excellent probe precisely because its relaxation is dominated by the well-understood quadrupolar mechanism [36], [64].

### Extensive ms Time-scale Motions in Lysate Ca^2+^-CaM but not in Lysate Ca^2+^-CaM-smMLCK(p)

The ligation of Ca^2+^ to the four metal binding sites in CaM is accompanied by significant conformational changes. These involve the majority of the methyl groups in the molecule that are displaced from the hydrophobic cores of the N- and C-terminal domains, relocalizing to surface exposed positions primed for binding target proteins [40]. Interestingly, ^1^H CPMG relaxation dispersion profiles of lysate Ca^2+^-CaM (10% trimmed mean Δ*R_ex_* = 17.0 s*^−^* ^1^) are significantly larger than for lysate apoCaM (10% trimmed mean Δ*R_ex_* = 8.5 s*^−^* ^1^), as illustrated for a number of residues in Figure 6A (compare brown *vs* green). The exchange process is wide-spread, affecting all but 2 of the methyl groups in CaM, Figure 6B, while by comparison, ms time-scale dynamics in lysate apoCaM are mostly confined to the C-terminal domain. Additionally, *k_ex_* = 1250±30 s*^−^* ^1^, from fits of lysate Ca^2+^-CaM dispersion profiles is double that determined for lysate apoCaM (600±55 s*^−^* ^1^). It is clear, therefore, that the exchange dynamics are very different between lysate apoCaM and lysate Ca^2+^-CaM; indeed this must be the case as metal exchange is much less prevalent in the Ca^2+^ loaded form of the protein (in the presence of excess Ca^2+^), while it is the major source of the broadening in lysate apoCaM. That the processes responsible for conformational heterogeneity in the two CaM states are very different is further shown in Figure 7 where Δ*R_ex_* values for lysate apoCaM and lysate Ca^2+^-CaM are poorly correlated. Finally, it is noteworthy that Akke and coworkers have measured much faster dynamics in a Ca^2+^ loaded-CaM C-terminal domain mutant using ^15^N *R_1ρ_* type of experiments (*k_ex_* ∼ 20 µs) that are linked to a transition between open and closed conformational states related to metal release [65].

The exposure of hydrophobic methyl containing side-chains upon addition of Ca^2+^ to CaM and the availability of a large number of native *E. coli* proteins in lysate that can potentially serve as weak, non-specific targets for Ca^2+^-CaM suggests that the pervasive ms fluctuations may derive from weak binding events. Indeed, Met side-chains play a pivotal role in substrate recognition [42], [51] and all of the Metε groups show exchange, with the largest Δ*R_ex_* values obtained for M124ε (72 s*^−^* ^1^) and M71ε (63 s*^−^* ^1^), respectively. If the exchange derives from a “frustrated” search process whereby Ca^2+^-CaM interacts with a range of non-cognate *E. coli* proteins then the ms dynamics is predicted to be quenched by addition of a high affinity target peptide, such as from the protein smooth muscle myosin light chain kinase (smMLCK). Peptide binding would sequester the hydrophobic methyl containing side-chains in a single conformation and hence eliminate exchange. Addition of smMLCK peptide (*K_d_* = 1 nM [51]) leads to a markedly improved ^13^C-^1^H correlation map (compare lysate Ca^2+^-CaM and lysate Ca^2+^-CaM-smMLCK(p), Figure 1B) and essentially eliminates ms timescale motions as detected via ^1^H CPMG relaxation dispersion experiments, Figure 8.

### Concluding Remarks

It is well known that the cellular milieu is very distinct from the highly controlled environment that is typically employed in “test-tube” studies of protein molecules. The large number of diverse biomolecules in the cell, including proteins, nucleic acids, lipids and metabolites and the high net concentration of protein molecules that typically greatly exceeds that used in *in vitro* biophysical studies can potentially lead to changes in protein structure and dynamics. Here we have presented a comparative study of methyl containing side-chain dynamics of CaM in apo, Ca^2+^-loaded and Ca^2+^-loaded peptide bound states dissolved in either buffer or *E. coli* lysate. Very similar ^13^C-^1^H methyl correlation spectra are obtained for apoCaM and Ca^2+^-CaM-smMLCK(p) in buffer and lysate indicating that the average structures of these states do not change with the different protein environments. By contrast, significant differences are observed in spectra of buffer and lysate Ca^2+^-CaM both in terms of linewidths and peak positions (Figure 1B) that likely reflect non-specific transient interactions between CaM and non-cognate protein partners in *E. coli* lysate. ^2^H spin relaxation experiments establish that ps-ns dynamics for apoCaM and Ca^2+^-CaM-smMLCK(p) are little affected by lysate, while the significant ms processes measured for lysate apoCaM and lysate Ca^2+^-CaM are absent in aqueous buffer. This study emphasizes both the similarities and the differences in protein dynamics between aqueous and in-cell like conditions and serves as a reminder that a complete understanding of protein structure and dynamics must ultimately take into account the unique cellular environment in which the protein functions.

## Materials and Methods

### Sample Preparation

BL21(DE3) pLysS *E. coli* cells (EMD Millipore) were transformed with a plasmid encoding CaM and were grown in deuterated M9 minimal media with 1 g/L ^15^NH_4_Cl and 3 g/L ^2^H,^12^C-glucose at 37°C. Production of U-[^2^H],Ile-δ1[^13^CHD_2_]-, Leu,Val-[^13^CHD_2_,^13^CHD_2_]-, Met[^13^CHD_2_]-labeled samples of CaM followed the protocol of Tugarinov *et al* with precursors added to the bacterial culture 1 hour before induction [66]. Over-expression of CaM was induced with 1 mM IPTG and proceeded overnight at 30°C. The resulting His-tagged protein was purified on a HisTrap HP column (GE Healthcare), followed by TEV protease cleavage of the His-tag and repurification over HisTrap HP and HiTrap Phenyl HIC HP columns (GE Healthcare). Purified CaM was buffer exchanged into the appropriate NMR buffer and concentrated using a 3 kDa MWCO centrifugal concentrator (Amicon). For apoCaM, the NMR buffer was 20 mM imidazole, pH 6.5 (uncorrected), 100 mM KCl, 1.5 mM EGTA, 100 µM NaN_3_ in 100% D_2_O. For Ca^2+^-CaM and Ca^2+^-CaM-smMLCK(p) samples, the buffer was 20 mM imidazole, pH 6.5 (uncorrected), 100 mM KCl, 6 mM CaCl_2_, 100 µM NaN_3_ in 100% D_2_O.

A smMLCK(p) peptide (NH_2_-ARRKWQKTGHAVRAIGRLSS-COOH) was purchased from GenScript (Piscataway, NJ) at >98% purity and was used without further purification. The peptide was resuspended in Ca^2+^-CaM NMR buffer. Dilute solutions of peptide and Ca^2+^-CaM were combined in a 1.25∶1 molar ratio and concentrated in a 3 kDa MWCO centrifugal concentrator to produce samples of Ca^2+^-CaM-smMLCK(p).

*E. coli* lysate was prepared by growing BL21(DE3) pLysS cells (EMD Millipore) in LB supplemented with chloramphenicol (34 µg/mL) at 37°C until OD_600_ ∼ 0.9. The cells were harvested, resuspended in D_2_O, lysed and the insoluble material spun down. 5 mM benzamidine, 0.1 mg/mL PMSF and 100 µM NaN_3_ were added to the clarified lysate, which was then filtered through a 0.22 µm syringe filter. Subsequently lysate was concentrated in a 3 kDa MWCO centrifugal concentrator. Total lysate protein concentration was determined by the BCA assay (Pierce) and samples of CaM in lysate were produced by diluting concentrated CaM and concentrated lysate to the appropriate volumes. All NMR samples were 1 mM CaM and lysate samples contained 100 g/L *E. coli* protein. Ca^2+^-CaM and Ca^2+^-CaM-smMLCK(p) lysate samples were supplemented with 6 mM CaCl_2_. The choice of 100 g/L *E. coli* protein represents a compromise between a ‘physiological’ amount of added protein (in cell protein concentrations range between 80 and 400 g/L) [67]–[69] and the ability to record very high quality spectra so that accurate relaxation data could be obtained. We estimate that reasonable quality data could be generated for lysate protein concentrations up to approximately 200 g/L.

Atomic absorption analysis was performed by the ANALEST facility of the Department of Chemistry at the University of Toronto on a 10 mg/mL sample of lysate. After correction for dilution, the following total metal concentrations were obtained: Ba, 0.004 mM; Ca, 0.292 mM; Cu, 0.001 mM; Fe, 0.292 mM; K, 31.203 mM; Mg, 1.300 mM; Mn, 0.035 mM; Na, 29.622 mM; Ni, 0.018 mM; and Zn, 0.093 mM. A 2× solution of these metals (excluding Ba and Cu) was prepared in D_2_O and mixed 1∶1 with apoCaM in D_2_O. EGTA was then added to 50 mM.

### NMR Spectroscopy

NMR spectra were recorded at 18°C on a Varian Inova spectrometer, 14.0T, equipped with a cryogenically cooled pulsed-field gradient triple resonance probe. Data sets were typically obtained with 72x512 complex points, acquisition times of 26x64 msec in *t_1_* and *t_2_*, respectively, and a 2.0 sec recycle delay. ^2^H *R_1_* and *R_2_* relaxation rates were measured using pulse schemes described previously [31], [32]. *R_1_* values were generated from 8 parametrically varied time points between 0.05 and 25 msec, while ^2^H *R_2_* rates were based on measurements of 8 to 11 time points between 0.05 and 15 msec for samples in aqueous buffer and 0.05 and 10 ms for samples in lysate. Total measuring times were approximately 36 and 20 hours for *R_1_* and *R_2_* data sets, respectively. ^2^H relaxation rates were subsequently obtained from fits to a single-exponential decay function, *Ae^−Rt^*, with errors calculated from the covariance matrix method [70]. ^1^H relaxation dispersion profiles were recorded using a pulse scheme described by Baldwin *et al*., [35] with a 30 msec constant-time CPMG relaxation delay and CPMG frequencies ranging between 33.3 and 2,000 Hz (18 or 32 hours for a data set comprising 18 2D spectra recorded on samples in buffer and lysate, respectively). All spectra were processed with NMRPipe/NMRDraw software [71] and peak intensities quantified with the program FuDA (http://pound.med.utoronto.ca/software.html) [70]. ^1^H relaxation dispersion profiles were calculated from the relation 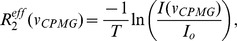 where *T* is the constant-time relaxation delay, ν*_CPMG_* = 1/(2δ) with δ the delay between successive refocusing pulses in the CPMG element and *I(ν_CPMG_)* and *I_o_* are peak intensities from spectra recorded with and without the delay *T*, respectively. Dispersion data were fitted using the program CATIA (http://pound.med.utoronto.ca/software.html) assuming a two-site global exchange model, as described previously [59], [72]. Initially methyl groups with Δ*R_ex_ >10* s*^−^* ^1^ were fitted together to obtain global values for *k_ex_* and *p_B_*. The global parameters were then fixed and all residues were subsequently fitted together to the two-site exchange model. A number of very small dispersion profiles (less than 3–4 s*^−^* ^1^) were observed in buffer CaM (from L105Cδ1/δ2, V91Cγ1 and Val55Cγ1) that are associated with an exchange process that is unrelated to the effects of lysate. These were removed prior to fitting.
